# Ultrathin Single‐Crystalline Boron Nanosheets for Enhanced Electro‐Optical Performances

**DOI:** 10.1002/advs.201500023

**Published:** 2015-05-05

**Authors:** Junqi Xu, Yangyang Chang, Lin Gan, Ying Ma, Tianyou Zhai

**Affiliations:** ^1^ State Key Laboratory of Material Processing and Die and Mould TechnologySchool of Materials Science and EngineeringHuazhong University of Science and Technology (HUST)Wuhan430074P. R. China

**Keywords:** boron, field‐effect transistors, field emission, photoelectricity, ultrathin nanosheets

## Abstract

Large‐scale single‐crystalline ultrathin boron nanosheets (UBNSs, ≈10 nm) are fabricated through an effective vapor–solid process via thermal decomposition of diborane. The UBNSs have obvious advantages over thicker boron nanomaterials in many aspects. Specifically, the UBNSs demonstrate excellent field emission performances with a low turn‐on field, *E*_to_, of 3.60 V μm^−1^ and a good stability. Further, the dependence of (turn‐on field) *E*_to_/(threshold field) *E*_thr_ and effective work function, *Φ*_e_, on temperature is investigated and the possible mechanism of temperature‐dependent field emission phenomenon has been discussed. Moreover, electronic transport in a single UBNS reveals it to be an intrinsic p‐type semiconductor behavior with carrier mobility about 1.26 × 10^−1^ cm^2^ V^−1^ s^−1^, which is the best data in reported works. Interestingly, a multiconductive mechanism coexisting phenomenon has been explored based on the study of temperature‐dependent conductivity behavior of the UBNSs. Besides, the photodetector device fabricated from single‐crystalline UBNS demonstrates good sensitivity, reliable stability, and fast response, obviously superior to other reported boron nanomaterials. Such superior electronic‐optical performances are originated from the high quality of single crystal and large specific surface area of the UBNSs, suggesting the potential applications of the UBNSs in field‐emitters, interconnects, integrated circuits, and optoelectronic devices.

## 1 Introduction

This is an open access article under the terms of the Creative Commons Attribution License, which permits use, distribution and reproduction in any medium, provided the original work is properly cited.

New appearing 2D structures, such as pure elements semiconductors graphene, silicene, phosphorene, and transition metal dichalcogenides (TMDs), their ultrathin thickness, and ultralarge specific surface area render them many unique properties and make them promising candidates of various applications, including field‐effect transistors (FET), sensors, energy storage, and so on.^1^, ^2^, ^3^, ^4^ Among these layered materials, pure element structures take an important place in 2D materials for their simple synthesis method and their controllable, excellent electronic properties.[[qv: 3a]] For example, graphene, a monolayer of carbon with a honeycomb lattice structure, is the first 2D material fabricated in the world. Nowadays, it is convenient to fabricate graphene through various methods such as exfoliation routes,^5^ chemical vapor deposition (CVD),^6^ carbon segregation,^7^ and epitaxial growth.^8^ Moreover, the modulation of electronic properties of graphene via doping^9^ or decoration^10^ has been demonstrated in many works. Consequently, a rising question is whether there are other types of pure element 2D materials waiting for exploration.^11^ Therefore, exploration of a new kind of pure elements 2D materials has certainly drawn considerable attention from both the fundamental and practical points of view.

Boron nanomaterials arouse enormous interest from both scientific and technological angles of view because of its unique chemical and physical properties, which ensure its wide applications in high‐temperature devices, high‐energy fuel, nuclear engineering, coatings, and field emission (FE).^12^ Intriguingly, boron has a band gap (≈1.5 eV),^11^,[[qv: 13d]] and the theoretical study predicted that it has also a high conductance (≈10^2^ Ω^−1^ cm^−1^) and carrier mobility (≈10^2^ cm^2^ V^−1^s^−1^),^14^ which guarantee it to be a perfect combination of ideal semiconductor properties for the applications in electronics and optoelectronics. Recently, a number of theoretical studies have also predicted the thermodynamically stable atomically thin 2D boron sheets,^15^ which are the analog of a single‐graphite sheet. Nevertheless, the low response to illumination and high turn‐on field in FE due to the substantial trap states in the amorphous layer phase seriously restrict their various optoelectronic applications.^16^ For instance, boron belt takes more than 72 h for both the rising process under illumination and the degradative process under darkness,[[qv: 16a]] combined with a low conductivity of ≈10^−3^ Ω^−1^ cm^−1^.^17^ Therefore, synthesis of high‐quality boron nanomaterials with high photoresponse and fast switching rates to illumination and favorable FE properties with low turn‐on field is essential. Single‐crystalline ultrathin boron nanosheets (UBNSs) can be expected as an ideal candidate of superior performances due to their anisotropic feature, free of trap states, large specific surface area, and the confinement of photon and carrier in 2D. Up to now, the fabrication of large‐scale, high‐quality, ultrathin BNSs is still a big challenge, and thus the reports on BNS‐based devices are quite limited.[[qv: 16a]],^17^

In this paper, single‐crystalline UBNSs were synthesized by an effective vapor–solid (VS) process via thermal decomposition of diborane (B_2_H_6_) under a lower pressure, higher temperature, and longer reaction time, compared with the current method.^18^ Different from previous boron nanobelt with thickness larger than 15 nm,^18^ here single‐crystalline boron NSs with only several nanometers in thickness have been reported first time. Accordingly, the FE properties of the UBNSs were studied for the first time, which exhibits a low turn‐on electric field of 3.60 V μm^−1^ at 10 μA cm^−2^, a higher current density of 7.47 mA cm^−2^ at 5.80 V μm^−1^, and a high field enhancement factor of 1363 at room temperature (RT). Furthermore, the temperature‐dependent FE measurements demonstrated that the temperatures greatly affect the turn‐on or threshold electric fields, emission current densities, and the effective work functions. Moreover, the field‐effect transistor (FET) measurement reveals that the UBNSs are p‐type semiconductors with carrier mobility of 1.26 × 10^−1^ cm^2^ V^−1^ s^−1^, two orders of magnitudes larger than current reported data.^19^ Besides, the temperature‐dependent transport mechanism was also discussed. In addition, the UBNSs photodetectors show a shorter response (response time and decay time of ≈2.4 and ≈3.6 s, respectively), a higher quantum efficiency of 1.78 × 10^5^%, and higher specific detectivity of 4.91 × 10^11^ Jones,[[qv: 16a]] compared to reported boron nanomaterials. These results suggest that the UBNSs are promising candidates to apply in both high performance field emission electron source and high‐speed photodetectors/photo‐electronic switches with high sensitivity.

## 2 Results and Discussion

### 2.1 The Morphology and Structure of the UBNSs

**Figure 1:**
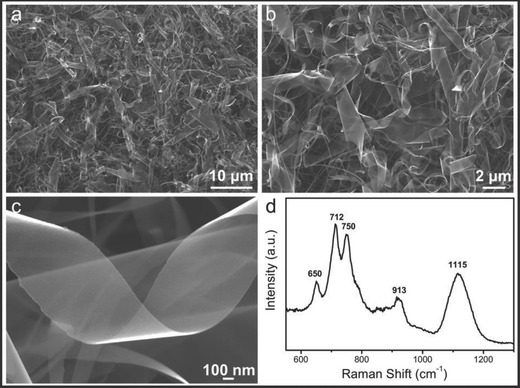
a,b) SEM pictures for the UBNSs at different magnifications; c) a typical image of enlarged UBNS; d) micro‐Raman spectrum of the UBNSs at RT.

The representative surface topographies of the synthesized UBNSs were imaged by field‐emission SEM (**Figure** **1**). The SEM images at different magnifications (Figure 1a,b) demonstrate that the UBNSs, with a width ranging from tens of nanometers to 3 μm and a length of ≈3–20 μm, uniformly grow over the silicon substrate. The nanosheets are smooth and almost transparent, which suggest that the BNSs are ultrathin (Figure 1c). The Raman spectrum identifies the nanosheets to be boron, as shown in Figure 1d. The main peaks are located at 650, 712, 750, 913, and 1115 cm^−1^, respectively, which coincide with previous literatures,[[qv: 13a]],^18^,^20^ and verify the formation of α‐tetragonal boron phase.

**Figure 2:**
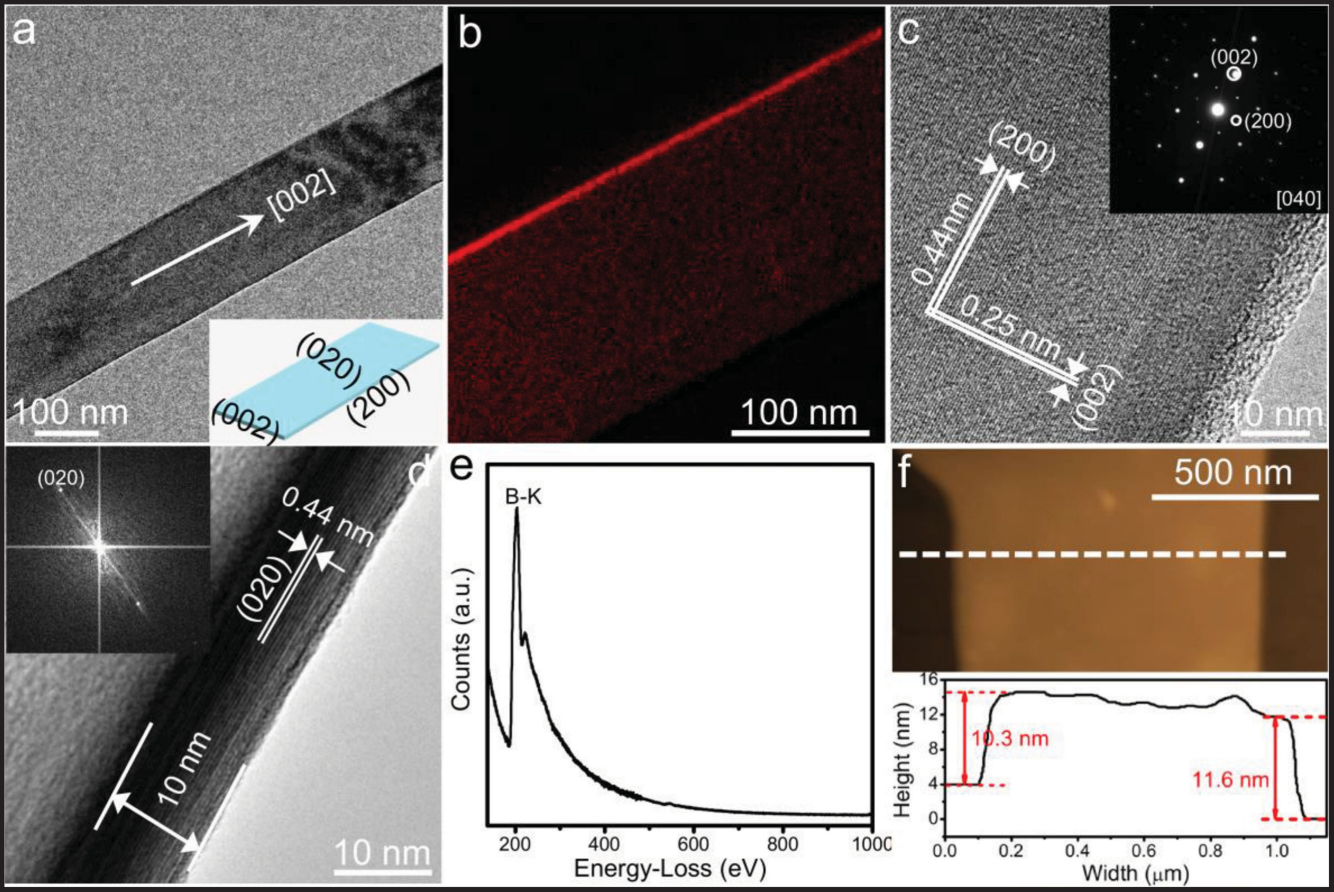
a) A single UBNS TEM picture, structural model, and their corresponding enclosing facets of the individual nanosheets (inset). b) Energy‐filtering mapping of boron element within the nanosheets. c) HRTEM picture and the corresponding SAED pattern (inset) revealing the [002] growth direction. d) HRTEM image and the corresponding SAED pattern (inset) showing the side facet. e) EELS spectrum acquired from the single UBNS. f) AFM picture (top) and the corresponding height profiles (bottom).

The detailed crystalline microstructure of the UBNSs was investigated by transmission electron microscopy (TEM). The low‐magnification TEM image of a typical individual NS is shown in **Figure** **2**a, which shows a smooth appearance and uniform width (≈180 nm). The elemental map displayed in Figure 2b further verifies the uniform distribution of boron throughout the whole nanosheet, consistent with the Raman spectrum. High‐resolution TEM observations (Figure 2c) and the selected‐area electron diffraction (SAED) pattern (inset of Figure 2c) further demonstrate the single‐crystalline nature, showing the clear interference fingers with two *d*‐spacings of ≈0.25 and 0.44 nm, respectively, corresponding to the (002) and (200) crystal faces of α‐tetragonal boron (JCPDS No. 75‐0218). Figure 2d is an HRTEM picture and the corresponding SAED pattern (inset) from the sideview of the UBNSs, which further confirms that the nanosheet is very thin (about 10 nm, ≈23 layers). Statistics data, based on a large number of TEM images (more than 50), reveal that the thicknesses of the nanosheets are ≈8–12 nm (although thinner boron nanosheets, 6.6 nm in thickness, can also be found in Figure S1). As shown in Figure 2e, the typical electron energy loss spectroscopy (EELS) from the UBNSs indicates that no oxygen, carbon, or other impurities are detected except the strong boron element characteristic K‐shell ionization edge (≈188 eV) signals, which is in good agreement with the boron nanostructures.^18^, ^21^ A typical atomic force microscope (AFM) picture of the UBNSs was shown on the top of Figure 2f, and the height profile (the bottom of Figure 2f) reveals that the edge height of the UBNSs is 10.3 (≈24 layers) nm and 11.6 nm (≈27 layers), respectively, which agrees with the HRTEM characterization well. All the results confirm the successful fabrication of high‐quality single‐crystal boron NSs with thickness about 10 nm oriented along the [002] direction (Figure 2a) and with (002), (200), (020) as enclosing facets (inset of Figure 2a). The growth of the UBNSs along the [002] direction is attributed to the (002) lattice planes of α‐tetragonal boron having the highest atom stacking density, and the total energy of the crystal would be greatly minimized through growing along this lattice direction. Comparing with the previously reported boron nanobelts, the UBNSs were synthesized in a higher temperature, lower pressure, slower gas flow, and longer reaction time, and thus are thinner and have better crystallinity.^18^ It is reasonable to expect that electron transport properties of the UBNSs would be benefited from its high quality of crystal and neglectable grain boundaries or other interfaces.^22^, ^23^

### 2.2 Field‐Emission (FE) Properties of the UBNSs

**Figure 3:**
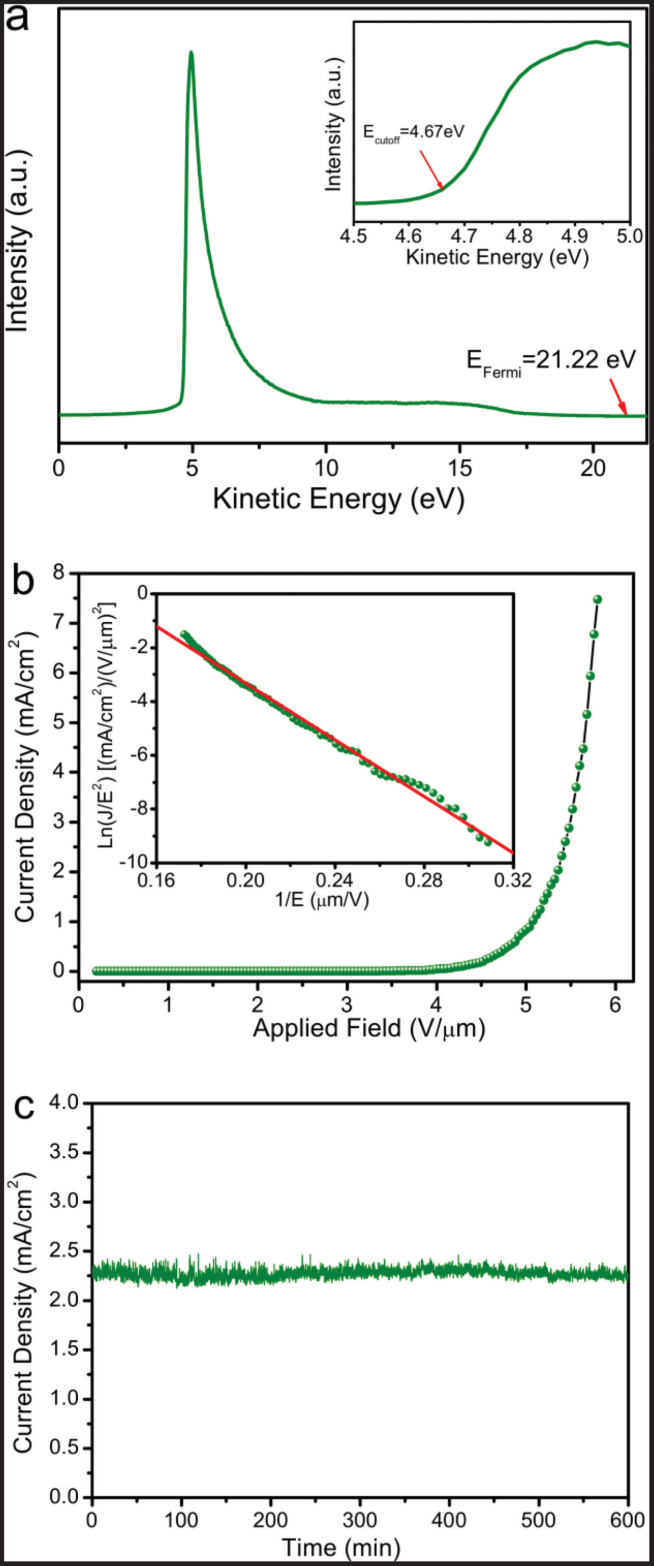
FE performances of the as‐synthesized UBNSs: a) UPS spectra of the as‐prepared UBNSs; the inset shows the magnified region near the low energy cut‐off energy; b) FE current density varies with the applied electric field (*J*–*E* plots) and the inset shows the corresponding Fowler–Nordheim curve (F–N plots) roughly revealing linear dependence; c) a typical FE stability curve of the UBNSs on Si substrates.

The ultrathin structure and good crystallinity suggest that the UBNSs are a potentially excellent field‐emission cathode. Interestingly, to the best of our knowledge, this work is the first study on boron nanosheet‐based field emission. We have employed ultraviolet photoemission spectroscopy (UPS) to measure the work function of the UBNSs. The full He I scan of the UPS spectrum of the films is shown in **Figure** **3**a. The *Φ* value is deduced to be 4.81 eV for the UBNSs (Derivation S1, see the Supporting Information), which is very close to boron bulk materials and nanostructures.[[qv: 12e]],^24^

In order to further explore the feasibility of applying the UBNSs in the vacuum microelectronic and nanoelectronic fields, the field‐emission characteristics were measured under a pressure of 8.0 × 10^−7^ Pa at RT (293 K). The evolution of field emission current density (*J*) with applied electric field (*E*) has been shown in Figure 3b. Notably, a turn‐on current density (10 μA cm^−2^) at a macroscopic field of as low as 3.60 V μm^−1^ and a high FE current density of 7.47 mA cm^−2^ at a macroscopic field of 5.80 V μm^−1^ can be read from Figure 3b. The results indicate that the UBNSs are ideal field emitters of high efficiency, which can surpass or comparable with B nanowires,[[qv: 12e]] LaB_6_/NdB_6_ nanorods,^25^ B_4_C nanowires,^26^ CdS nanobelts,^27^ MoS_2_ sheets,^28^ and graphene.^29^

The FE current–voltage characteristics are further studied by the Fowler–Nordheim (F–N) Equation (1), 30

(1) $$J=A\frac{\beta^{2}E^{2}}{\phi }\exp\frac{-B\phi^{3/2}}{\beta E}$$

where *A* and *B* are the constants with values of 1.54 × 10^−6^ A eV V^−2^ and 6.83 × 10^3^ eV^−3/2^ V μm^−1^, respectively; *J*, *E*, and *β* represent the current density, the applied field, and the field enhancement factor, respectively; *Φ* is the work function of the material (4.81 eV for the UBNSs). Generally, the *β* are usually associated with the emitter geometry, alignment, crystal structure, and spatial orientation of distribution of emitting centers. For instance, the FE performance of LaB_6_ nanowires has been proved to be dramatically improved by sharpening the emitter tip, increasing their aspect ratios, spatial uniformity, and alignment (arrays vertical to the substrates).^31^ From the F–N slope in the inset of Figure 3b, the field‐enhancement factor was calculated to be as high as 1363. Such good properties may be attributed to ultrathin structure and high length‐to‐thickness ratios (length of dozens of micrometers and thickness of only 10 nm, resulting in the high ratios of 10^5^–10^6^). Moreover, the FE stability measurements of the UBNSs (Figure 3c) show that the UBNSs exhibit a constant emission of ≈2.3 mA cm^−2^ under an applied field of 5.40 V μm^−1^ and the low current fluctuations of 7.6% during 10 h. Such good stability on emission current promises the potential of utilizing the UBNSs in field emitters.

**Figure 4:**
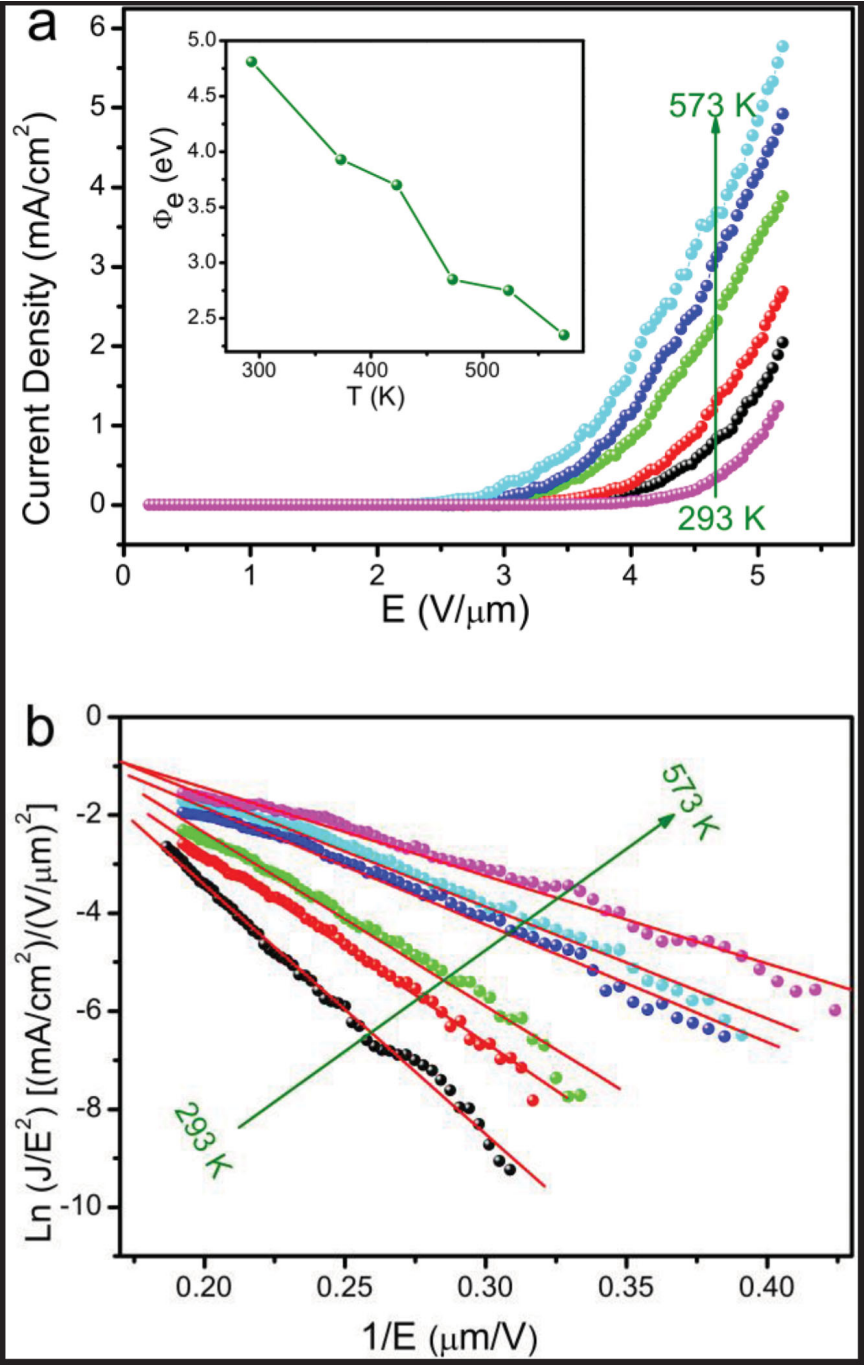
a) *J*–*E* plots under different temperatures and their corresponding effective work functions (inset); b) the corresponding F–N plots.

Temperature of the cathode has a great impact on the FE characteristics of material.^32^ **Figure** **4**a demonstrates the relation of the applied electric field to the emission current density for the UBNSs at temperatures of 293, 373, 423, 473, 523, and 573 K, respectively. The turn‐on fields drop obviously from 3.60 to 2.33 V μm^−1^, the threshold fields (at an emission current density of 1 mA cm^−2^) reduce dramatically from 5.08 V μm^−1^ to 3.72 V μm^−1^, whereas the emission current density rises remarkably from 1.24 to 5.77 mA cm^−2^ (at the electrical field of 5.16 V μm^−1^) upon increasing the temperatures from RT to 573 K, as listed in **Table** **1**. The corresponding F–N curves, plotted as ln(*J*/*E^2^*) versus 1/*E*, are depicted in Figure 4b. The emitting electrons can mainly be attributed to field emission effects, supporting by those straight line relation, which is also verified by previous literatures.^32^, ^33^ Theoretically, the F–N model predicts that FE properties are solely determined by the work function (*Φ*) and field‐enhancement factor (*β*).^30^ In fact, it is widely accepted that the *β* (1363) exhibits a neglectable fluctuation with temperature; therefore, the effective work function (*Φ*_e_) under different temperatures can be deduced from the relation *k_T_* = −*BΦ*_e_^3/2^/*β* (*k_T_* is the F–N slope at temperature *T*).^34^ As depicted in the inset of Figure 4a, the effective work function (*Φ*_e_) decreases from 4.81 to 2.35 eV with the increase of temperature, which explains well the obvious decrease of the turn‐on field and the significant increase of emission current with temperature increasing.

**Table 1:** Key parameters of FE performance for the UBNSs at different temperatures

Temperature [K]	Turn‐on field [V μm^−1^]	Threshold field [V μm^−1^]	F–N slope	FE current at 5.16 V μm^−1^ [mA cm^−2^]	Effective work function [eV]
293	3.60	5.08	52.62	1.24	4.81
373	3.28	4.81	38.85	2.04	3.93
423	3.12	4.56	35.44	2.68	3.70
473	2.60	4.12	23.96	3.89	2.85
523	2.56	3.91	22.75	4.92	2.75
573	2.33	3.72	17.92	5.77	2.35

**Figure 5:**
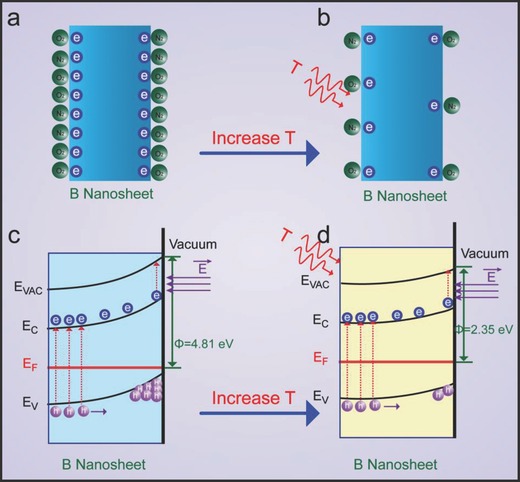
a,b) Sketch of the UBNSs under heating; c,d) illustration of the UBNSs band diagrams under heating.

With regard to the origin of temperature‐dependent FE characteristics, there are four mainstream opinions. First, the rising of the Fermi level (*E*_F_) with temperature lowers the effective work function (*Φ*_e_).[[qv: 32a]],^34^ However, the simulation reveals that the work function *Φ*_e_ is increasing from 4.81 to 4.83 eV (Derivation S2, see Supporting Information), which contradicts with the FE data shown in the inset of Figure 4a. Consequently, in the case of boron nanosheets, it is inappropriate to ascribe the enhanced FE current to the rising of the Fermi level. Second, the increasing enhancement factor (*β*) lowers the effective work function.[[qv: 32b]],^35^ In fact, upon the temperature increasing, no physical facts support the rising of *β*, so the enhancement factor should be a constant.^31^ Third, thermionic electrons emission improves the current. However, thermionic current is negligible below 1000 K in comparison with FE current.[[qv: 32b]],^36^ Fourth, gas desorption lowers the effective work function. Recently, Li et al.^37^ reported the enhanced FE properties and ascribed this feature to the oxygen molecules desorbed from the surface of the graphene sheets with the temperature increasing; Deng et al.^38^ investigated the FE temperature of CNTs and attributed this characteristic to the oxygen desorption; Hsu et al.^39^ found the enhanced FE effects under the UV light irradiation and attributed the behavior to the oxygen desorption under illumination. Therefore, after excluding the former three reasons, the forth mechanism about gas molecular desorption may be favorable to the present experiment results.^31^, ^40^ In other worlds, the work function of the UBNSs may be decided by the adsorption or desorption of gases on the surface. Based on that, we propose the following model (as shown in **Figure** **5**) to explain the enhanced field emission effects with the rise of temperature. At room temperature, for the reason of large specific area of the UBNSs, the gas molecules^41^ (such as O_2_ and N_2_, etc.) adsorbed on the UBNSs surface capture free electrons from the UBNSs, which forms a depletion layer on the UBNSs surface (Figure 5a) and then results in band bending (Figure 5c). When the temperature was rising from RT to 573 K, the adsorbed gases were released from the surface (Figure 5b) of UBNSs surface by the thermal energy, which encouraged the curved band to be more flat (Figure 5d). As a result, the further flat band would lower the work function, which lowers the turn‐on voltage and improves the FE current, consistent with the experimental results at different temperatures. The adsorption/desorption effect plays an important role in temperature‐dependent FE characteristics, similar to the phenomena in ZnO nanowires^39^ and LaB_6_ nanostructures.^31^, ^40^

### 2.3 Electrical Transport and Photoconductive Properties of the UBNSs

**Figure 6:**
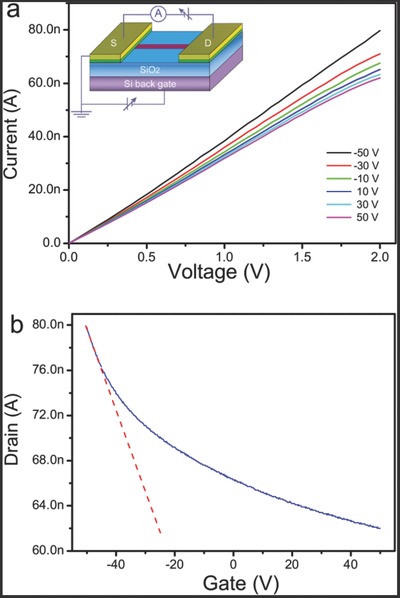
Electronic characterization of fabricated UBNS FET device: a) *I*_sd_–*V*_sd_ plots at different gates (*V*_g_), and schematic of single UBNS (inset); b) *I*_sd_–*V*_g_ plots.

Boron has a theoretical high conductivity (≈10^2^ Ω^−1^ cm^−1^) and carrier mobility (≈10^2^ cm^2^ V^−1^s^−1^); however, previous reported results showed much lower conductivity (10^−3^−10^−7^ Ω^−1^ cm^−1^),^17^, ^42^ and lower carrier mobility (10^−2^−10^−8^ cm^2^ V^−1^ s^−1^)^19^, ^43^ due to the poor quality of the boron structures. These issues have hindered its applications in electronics and optoelectronics. The UBNSs are expected to have better electronic properties for its better crystallinity and large specific surface area. The conductivity of the UBNSs was calculated to be 2.8 × 10^−2^ Ω^−1^ cm^−1^ in vacuum at RT (Figure S2), which is the highest value among reported results of pure bulk boron, boron nanowires, and nanobelts devices.^17^, ^42^ We attribute the improvement of conductivity to the ultrathin thickness, the high single crystalline, and large contact area between NS and electrodes. To investigate the electronic characteristics of the UBNSs, a field‐effect transistor based on a single UBNS has been fabricated on a silicon wafer with 500 nm thick SiO_2_ on the top (inset of **Figure** **6**a), and its output curve has been shown in Figure 6a. The current increases monotonously in the gate voltage range of 50 V to ≈50 V, suggesting that the UBNSs are p‐type semiconductors in accord with boron nanowires and bulk boron.^19^, ^44^, ^45^ The transfer characteristic (*I*_SD_–*V*_g_) of FET at *V*_SD_ = 2 V is depicted in Figure 6b, and the estimated carrier mobility is ≈*μ* = 1.26 × 10^−1^ cm^2^ V^−1^ s^−1^ (Derivation S3, see Supporting Information). This observed mobility is about two orders of magnitudes of that of nanowires, and also higher than that of pure bulk boron.^19^, ^43^

**Figure 7:**
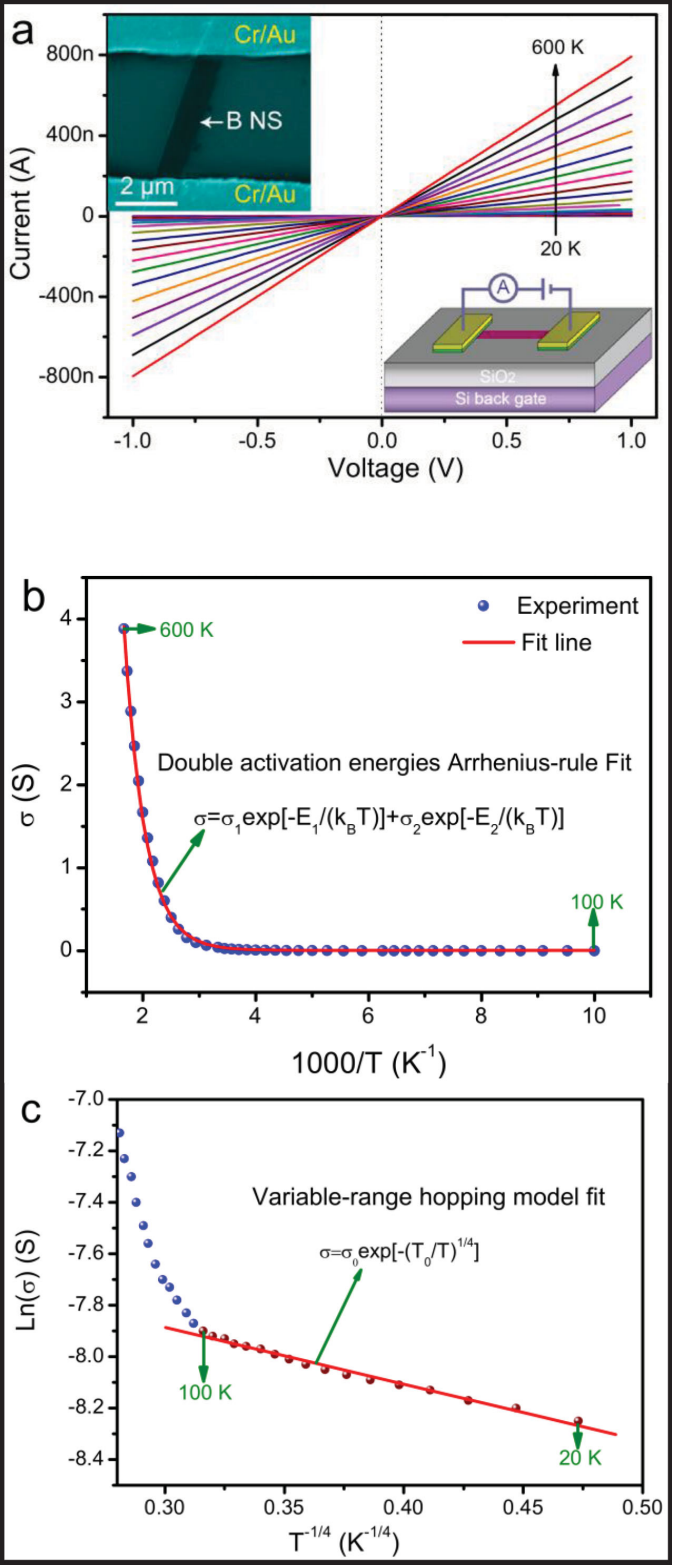
a) The current versus voltage (*I*–*V*) plots for single UBNS at different temperatures (20–600 K); the insets show an SEM picture (top) of single nanosheet device and schematic illustration (bottom); b) *σ*–*T* ^−1^ plot between 100 and 600 K; the red line is the fitting curve based on Equation (2); c) Ln(*σ*)–*T* ^−1/4^ between 20 and 100 K; the red line is the fitting curve based on Equation (3).

In order to investigate the transport mechanism of the UBNSs, the DC resistivity of single UBNS was measured under different temperatures. The insets of **Figure** **7**a show an SEM image (top) of a single UBNS‐based device and a schematic illustration (bottom). The current–voltage curves of the device in the temperature range from 20 to 600 K in vacuum are presented in Figure 7a, from which we can deduce that the UBNSs exhibit a typical semiconducting behavior because the current increases with rising temperature. From the characteristics of ln(*σ*)–1000/*T*, the whole temperature range can be divided into two temperature regions: a high‐temperature region (100 K < *T* < 600 K) and a low‐temperature region (20 K < *T* < 100 K). In the high *T* region, the measured conductivity *σ* of the UBNSs as a function of 1000/*T* is shown in Figure 7b, which exhibits a typical Arrhenius feature originated from the thermal activation conduction processes. Temperature‐dependent conductivity can be well quantitatively described by the double activation energies equation^46^

(2) $$\sigma =\sigma_{1}\exp\left(-\frac{E_{1}}{k_{B}T}\right)+\sigma_{2}\exp\left(-\frac{E_{2}}{k_{B}T}\right)$$

where *σ*_1_ and *σ*_2_ are the prefactors of conductance, *E*_1_ and *E*_2_ are two kinds of activation energies during the thermal activation conduction processes. The red line in Figure 7b is the fitting curve (fitting parameters are shown in Table S1, Supporting Information) based on Equation (2), which matches well with the experimental data. The dual‐activation energies characteristic may be a common phenomenon in boron‐based material.^47^ Although there is a little deviation in activation energies between different UBNSs samples (more than 20 samples have been studied), the temperature‐dependent conductivity curves exhibit almost the same Arrhenius features. This might be explained by the fact that the acceptor carriers (holes) were transported to the valance band due to the thermal activation.^48^ Then, the *E*_1_ (233 meV) mode and *E*_2_ (17 meV) mode are related to the deep acceptor states and the shallow acceptor states, respectively. No intentional doping for holes is carried out in our experiments; thus, the origin of holes is unclear at present. One probable explanation might be related to the excess oxygen,[[qv: 16a]] which has two possible models: one is cation vacancy and the other is interstitial oxygen. Oxygen playing an important role in the origin of positive holes can also be found in other p‐type semiconductors, such as CuAlO_2_ 48 and Cu_2_O films.^49^

In the low‐*T* region, the estimated activation energy is around 0.8 meV if the experimental data (20 K < *T* < 100 K) are also fitted with the Arrhenius law. However, the fitted activation energy is unreasonably low, which indicates that the conductivity of UBNSs at the low‐temperature region is dominated by a complete different conduction transport mechanism. It is generally accepted that at a low temperature region, temperature‐dependent conductivity follows the Mott variable‐range hopping (VRH) model^46^, ^50^

(3) $$\sigma =\sigma_{0}\exp\left[-{\left(\frac{T_{0}}{T}\right)}^{1/4}\right]$$

where *σ*_0_ is the prefactor of conductance and *T*_0_ is a constant. Figure 7c depicts the relation between ln(*σ*) and (1/*T*)^1/4^ in the low‐temperature region (20 K < *T* < 100 K), and the red line in Figure 7c shows the fitting curve (fitting parameters are shown in Table S1, Supporting Information). The good linear dependence magnifies that the Mott's VRH conduction mechanism dominates the electronic conduction behavior in this temperature region. In short, there are two different mechanisms playing roles in the whole testing temperature range, that is, the Arrhenius thermal activation mechanism dictates the conduction behavior in the high‐temperature region (100 K < *T* < 600 K) and Mott's variable‐range hopping mechanism dominates the low‐temperature region (20 K < *T* < 100 K). Actually, such a manifestation (multiple conduction mechanisms coexistence) was also discovered in other materials such as V_2_O_5_ nanowires,^50^ CuAlO_2_ films,^48^ and ZnO films.^46^

**Figure 8:**
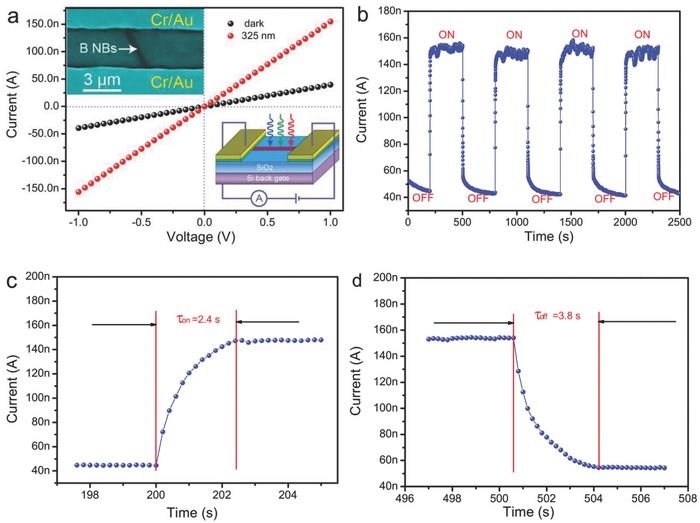
a) The *I*–*V* curves for UBNS photodetector illuminated under 325 nm light and dark condition; the insets show SEM picture (top) of UBNS device and schematic illustration (bottom); b) the on/off behavior of the UBNS photodetector illuminated under 325 nm light; c) the enlarged portion of the rise time; d) the enlarged portion of the decay time.

The high crystallinity of the UBNSs, combined with a band gap of 1.5 eV, benefits the UBNSs to be applied in optoelectronics and microelectronics. As illustrated in the bottom inset of **Figure** **8**a, Cr/Au parallel electrodes (10 nm/100 nm) were deposited on the nanosheets dispersed on a SiO_2_/Si substrate by using a standard lithography procedure. The uncovered part of the nanosheet, i.e., channel of device was exposed to the incident light. The SEM image of the single UBNS‐based device is shown in the upper inset of Figure 8a. Typical *I*–*V* curves of a single device, measured under the dark condition and under 325 nm light illumination (17.9 mW cm^−2^), are presented in Figure 8a. Ohmic contacts between the nanosheet and the Cr/Au electrodes can be observed, supported by the linear behavior of the *I*–*V* curves. Different from Schottky contact that is usually observed in nanoparticle/nanowire‐based photodetectors,^51^ the Ohmic contact ensures that there are no photo‐induced carriers trapped or blocked at the interface between the Cr/Au electrodes and the UBNSs. A significant improvement of current (155.9 nA) can be detected when the device was illuminated by 325 nm light, which is approximately four times the dark current (39.3 nA). The considerable improvement in photocurrent results from the formation of electron–hole pairs induced by the incident illumination and the large surface to incident illumination.^52^ Figure 8b shows the time‐dependent photoresponse curve of the UBNSs photodetector recorded by alternately switch on/off 325 nm light under a bias of 1.0 V. It can be seen that the four cycles of current under light “on” and “off” demonstrate inconsiderable difference that falls in the noise level, indicating the good reversibility and high stability of the UBNSs optical switches over operational time duration. The enlarged portions correspond to photocurrent rising (Figure 8c) and decaying (Figure 8d) processes, and suggest that the rise and decay time were 2.4 and 3.6 s, respectively. Hitherto, only two papers involved the photodetection of B‐based nanostructures.[[qv: 16a]],^53^ Specifically, an amorphous boron nanowire photodetector exhibits the rise time of 10 s and the decay time of 20 s.^53^ Worse, the rising time of 3 d and the decay time of 3.7 d were observed in a boron nanobelt photodetector even at a bias of 2 V.[[qv: 16a]] These poor photoresponse are usually related to the abundant trap states in the bulk/surface amorphous phase.[[qv: 16a]] It is worthwhile noting that the UBNSs high photosensitivity, quick photoresponse, and reliability are attributed to its high crystallinity of single crystal,^22^, ^54^ large specific surface area exposed to light,^52^ free of trap states,[[qv: 16a]] and the lower recombination barrier.^55^

Besides the response time, there are other three critical parameters for the evaluation of a photodetector performance. 1) Responsivity *R_λ_*, which evaluates the response of the photocurrent to incident optical power on the active area; 2) external quantum efficiency *EQE*, which calculates the numbers of electron–hole pairs detected per incident photon; 3) specific detectivity D*, one of the typical values used to evaluate the performance of a detector, which measures a photodetector using the unit of cm Hz^1/2^ W^−1^ (also called Jones). Based on the present experimental results, the values of *R_λ_*, EQE, and D* of the UBNSs are 465 A W^−1^, 1.78 × 10^5^%, and 4.91 × 10^11^ Jones, respectively (Derivation S4, see Supporting Information). These values are the first report for boron nanostructure‐based nanodevices and also much higher/or comparable to other semiconductor photodetectors.[[qv: 55a]],^56^ The above‐mentioned results suggest that UBNSs have great potential applications in nanoscale photodetectors and photoelectronic switches of high sensitivity and high speed.

## 3 Conclusions

In summary, large‐scale and high‐quality single‐crystalline UBNSs were fabricated through an improved VS process method. HRTEM and SAED show that the boron nanosheets have an α‐tetragonal structure with good crystallinity orientated along the [002] direction. Field emission properties of the UBNSs have been investigated first time, which showed a low turn‐on electric field of 3.60 V μm^−1^, a low threshold electric field of 5.08 V μm^−1^, a high electric field enhancement factor of 1363, and a high current density of 7.47 mA cm^−2^ at 5.80 V μm^−1^. Moreover, the FET measurement demonstrated that the UBNSs showed an intrinsic p‐type semiconductor behavior with the carrier mobility of 1.26 × 10^−1^ cm^2^ V^−1^ s^−1^. The electronic transport can be explained by double thermal activation processes at high temperature (100 K < T < 600 K) and Mott's variable‐range hopping process at low temperature (20 K < T < 100 K). In addition, the UBNSs exhibit a fast, reversible, and stable photoresponse. Besides, a high photoconductive gain (465 A W^−1^), a high quantum efficiency of 1.78 × 10^5^%, and a high specific detectivity of 4.91 × 10^11^ Jones were also observed. The high photosensitivity and fast photoresponse result from the high crystallinity of the UBNSs, large specific surface area exposed to light, free of trap states, and lower recombination barriers in the single‐crystalline UBNSs. These results reveal that the UBNSs have great potential applications in electronics and optoelectronics.

## 4 Experimental Section

*Synthesis and Characterization of UBNSs*: The synthesis of UBNSs was conducted by a catalyst‐free VS process in a 30 mm external‐diameter quartz tube placed inside as shown in Figure S3 (see the Supporting Information). In brief, several pieces of cleaned silicon wafers were located at upstream as substrates for deposition. Before the heating process, the quartz tubes were first pumped to low pressure ≈0.5 Pa and then washed with a mixed gas (5% H_2_ + 95% Ar, volume percent calculation), and this process was repeated for three times to remove oxygen in the system completely. Then, the system was heated to a target temperature, 950 °C, with a temperature rising rate of ≈15 °C min^−1^ under pumping vacuum. A steady mixed gas (5% B_2_H_6_ + 95% Ar) with a flow rate of 5 sccm was introduced into the system for 120 min, and then the reaction pressure indicated as ≈8 Pa. After that, the furnace was powered off and the temperature was dropped down to RT naturally.

The UBNSs were characterized by a field‐emission scanning electron microscope (SEM, S4800, Hitachi; JSM‐7600F, JEOL), a transmission electron microscopy (TEM, Tecnai G2 F30 S‐TWIN, FEI), and an atomic force microscopy (AFM, SPM9700, Shimadzu). The Raman spectra were recorded by a laser Raman spectrometer (LabRAM HR800, Horiba JobinYvon) using 532 nm Ar ion laser as the excitation source. Ultraviolet photoelectron spectroscopy (UPS) was measured using an ESCALab 250Xi from Thermo Scientific under a vacuum of ≈6.0 × 10^−6^ Pa. The field‐emission properties were conducted at different temperatures based on a home‐built parallel‐plate FE system in a system (8.0 × 10^−7^ Pa).

*Device Fabrication and Characterization*: To fabricate a single‐nanostructure device, the UBNSs were dispersed in ethanol and then deposited on the surface of SiO_2_ (500 nm)/Si(P^+^). The standard photolithography technique (MDA‐400M, Midas) followed by Cr/Au (10 nm/100 nm) electrodes deposition with EB evaporation (Nexdep, Angstrom Engineering) and a standard lift‐off procedure were adopted here. The current–voltage characteristics were monitored by a low‐temperature cryogenic probe station (CRX‐6.5K, Lake Shore) and a semiconductor parameter analyzer (4200‐SCS, Keithley).

For photodetector measurement, all the measurements were conducted in atmospheric conditions and the photocurrent was collected by a semiconductor parameter analyzer (4200‐SCS, Keithley). A He–Cd laser (IK3301R‐G, Kimmon) was focused and guided on the NS device perpendicularly. To measure the time response of the B NS device to light irradiation, a mechanical chopper was used to turn on and off the light irradiation. The light intensity was calibrated by using a UV‐enhanced Si photodiode.

## Supporting information

As a service to our authors and readers, this journal provides supporting information supplied by the authors. Such materials are peer reviewed and may be re‐organized for online delivery, but are not copy‐edited or typeset. Technical support issues arising from supporting information (other than missing files) should be addressed to the authors.

Supplementary
