# Supplementary material for: Ultrathin Single‐Crystalline Boron Nanosheets for Enhanced Electro‐Optical Performances
Source: Adv Sci (Weinh). 2015 May 5;2(6):1500023. doi: 10.1002/advs.201500023 (PMC5115407; doi:10.1002/advs.201500023)
Supplement: Supplementary file 1 — Supplementary [file ADVS-2-0h-s001.pdf]

Copyright WILEY-VCH Verlag GmbH & Co. KGaA, 69469 Weinheim, Germany, 2015.

ADVANCED SCIENCE
Open Access

Supporting Information

for *Adv. Sci.*, DOI: 10.1002/advs. 201500023

Ultrathin Single-Crystalline Boron Nanosheets for Enhanced Electro-Optical Performances

*Junqi Xu, Yangyang Chang, Lin Gan, Ying Ma, and Tianyou Zhai**

Supporting information

Ultrathin Single-Crystalline Boron Nanosheets for Enhanced Electro-Optical Performances

*Junqi Xu, Yangyang Chang, Lin Gan, Ying Ma, and Tianyou Zhai**

Table S1. The fitting parameters of the equation (2) and equation (3)

σ_1 (S)	σ_2 (S)	E_1 (meV)	E_2 (meV)	σ_0 (S)	T_0 (K)
3.5327×10^{2}	2.4811×10^{-3}	233	17	7.2844×10^{-4}	2.3687×10^{1}

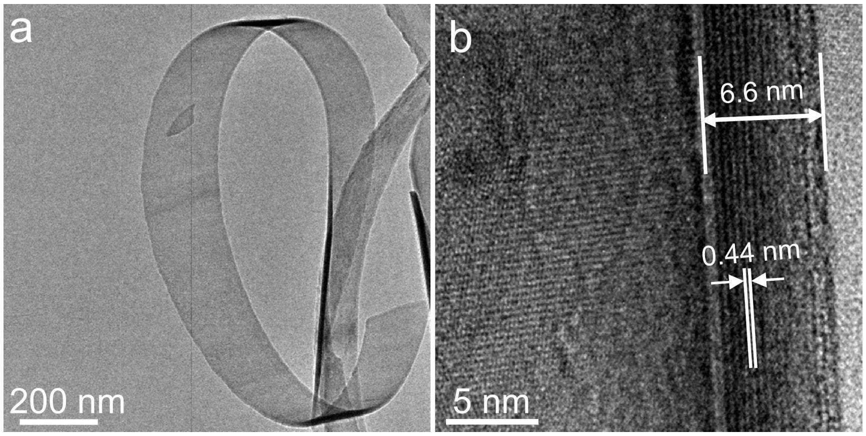

Figure S1. (a) TEM images of the UBNSs; (b) HRTEM image verifying that the nanosheet is very thin.

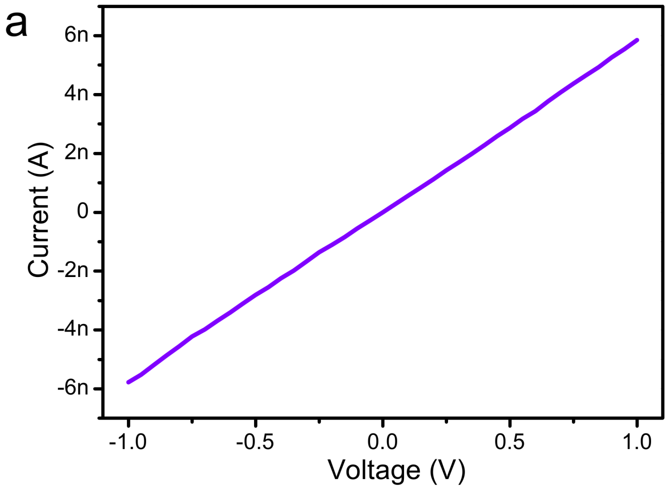

Figure S2. A typical I-V curve of the UBNSs in vacuum at RT

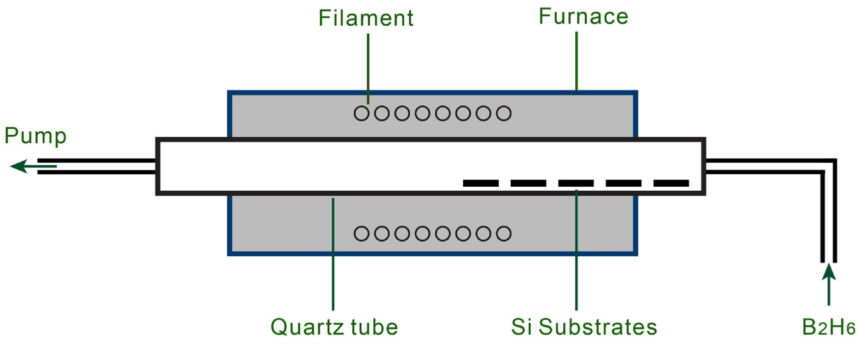

Figure S3. A schematic illustration of the CVD growth for the UBNSs.

Derivation S1. Calculation of the work function φ

The work function (φ) of the UBNSs can be calculated through the famous Einstein photoelectric effect equation:

$$h\nu - \phi = E_{Fermi} - E_{cutoff}$$

Where $h\nu$ is the radiation energy of the He I line, ϕ is the work function of material, E_{Fermi} is the Fermi energy, E_{cutoff} is the secondary electron emission edge. Here, $h\nu$=21.22 eV, E_{Fermi}= 21.08 eV, E_{cutoff}=4.67 eV, then ϕ=4.81 eV.

Derivation S2. Change of the effective work function φ_e

The boron NSs is a *p*-type semiconductor and the effective work function (φ_e) can be expressed as:

$$E_F = \frac{E_c + E_v}{2} + \frac{3}{4} k_B T \ln(\frac{m_p}{m_n})$$

$$\phi_e = E_{vac} - E_F$$

Where, E_F, E_c and E_v represent the Fermi energy, the conduction band energy and the valence band energy, respectively; m_n and m_p represent the effective mass of electron and hole, respectively; ϕ_e is the effective work function; k_B is the Boltzmann constant. Here, $m_n = 4.5m_0$, $m_p = 1.8\,m_0$, $k_B = 1.38\times10^{-23}\mathrm{JK}^{-1}$, E_{vac}=0. Then the above equations can be expressed as:

$$E_F = \text{-}4.7927 - 0.59\times10^{-4}\times T$$

$$\phi_e = -E_F$$

Base on numerical simulation, we can draw the conclusion that the E_F is lowering from -4.81 to -4.83 eV; meanwhile the work function ϕ_e is increaing from 4.81 to 4.83 eV upon the temperature increasing from RT to 573 K.

Derivation S3. Estimation of carrier mobility μ

The electron mobility (μ) can be calculated from the following equations:

$$g_m = \left.\frac{dI_D}{dV_G}\right|_{V_{SD}=const}$$

$$C_g = \frac{\varepsilon_r \varepsilon_0}{h_o}$$

$$\mu = \frac{L_{ch} g_m}{W_{ch} C_g V_{DS}}$$

Where g_m, μ and C_g represent the transconductance, electron mobility and gate capacitance, respectively; L_{ch}, W_{ch} are the length and width of the B NSs FETs, respectively; ε_0, ε_r and h_0 are the vacuum dielectric permittivity, relative dielectric permittivity, the thickness of the dielectric layer, respectively. Here, L_{ch}=4.3 μm, W_{ch}=0.9 μm, ε_0=8.85×10^{-12} Fm^{-1}, ε_r=3.8 h_0=500 nm. The g_m can be calculated to be 3.54×10^{-10} A V^{-1} from the gradient of transfer characteristic curve**.** Then,

$$C_g = \frac{\varepsilon_r \varepsilon_0}{h_o} = \frac{3.8 \times 8.85 \times 10^{-12} F / \mathrm{m}}{500 \times 10^{-9} m} = 6.73 \times 10^{-5} F\,\mathrm{m}^{-2};$$

$$\mu = \frac{4.3 \mu m \times 3.54 \times 10^{-10} A / V}{0.9 \mu m \times 6.73 \times 10^{-5} F / \mathrm{m}^2 \times 2V} = 1.26 \times 10^{-5} m^2 / (sV) = 1.26 \times 10^{-1} cm^2 V^{-1} s^{-1}$$

Therefore, the estimated carrier mobility (μ) of B NSs is ca. 1.26×10^{-1} cm^2 V^{-1}s^{-1}.

Derivation S4. Calculation of R_λ, *EQE* and *D**

The responsivity (R_λ), external quantum efficiency (*EQE*) and specific detectivity (*D**) can be calculated from the following equations:

$$R_\lambda = \frac{\Delta I_\lambda}{P_\lambda S}$$

$$EQE = \frac{hcR_\lambda}{e\lambda}$$

$$D^* = \frac{R_\lambda S^{1/2}}{(2eI_d)^{1/2}}$$

Where, ΔI_λ, P_λ, S and I_d represnt the photocurrent, light power, effective area of active material, and dark current, respectively; the parameters h, c, e and λ are the Plank constant, light speed, charge of electron, and wavelenght of incident light, respectively. Here, ΔI_λ=116.6×10^{-9} A, P_λ=17.9×10^{-3} W cm^{-2}, S=1.4×10^{-8} cm^2, I_d=39.3×10^{-9} A, h=6.6×10^{-34} Js, c=3.0×10^8 ms^{-1}, e=1.6×10^{-19} C, λ=325×10^{-9} m, then

$$R_\lambda = \frac{\Delta I_\lambda}{P_\lambda S} = \frac{116.6\times10^{-9}A}{17.9\times10^{-3}W/cm^2\times1.4\times10^{-8}cm^2} = 4.65\times10^2\,AW^{-1}$$

$$EQE = \frac{hcR_\lambda}{e\lambda} = \frac{6.6\times10^{-34}Js\times3.0\times10^8 m/s\times4.65\times10^2 A/W}{1.6\times10^{-19}C\times325\times10^{-9}m} = 1.78\times10^3 = 1.78\times10^5\%$$

$$D^* = \frac{R_\lambda \mathrm{S}^{1/2}}{(2eI_d)^{1/2}} = \frac{465A/W\times(1.4\times10^{-8}cm^2)^{0.5}}{(2\times1.6\times10^{-19}\,\mathrm{C}\times39.3\times10^{-9}A)^{0.5}} = 4.91\times10^{11}cm\,\mathrm{Hz}^{1/2}\,W^{-1} = 4.91\times10^{11}\,\mathrm{Jones}$$

Therefore, the calctulated R_λ, *EQE* and *D** are 4.65×10^2 AW^{-1}, 1.78×10^5%, and 4.91×10^{11} Jones.
